# Phage enabled precision drug delivery: dual function platforms for therapeutics and genetic cargo transport

**DOI:** 10.3389/fmicb.2026.1812871

**Published:** 2026-05-05

**Authors:** Md. Sharifull Islam, Jie Fan, Marshia Ahmed Vabna, Nabila Haque, Sawda Binte Monir, Yikui Li, Ishatur Nime, Mrityunjoy Acharjee, Fan Pan

**Affiliations:** 1 Center for Cancer Immunology, Institute of Biomedicine and Biotechnology, Shenzhen Institute of Advanced Technology (SIAT), Chinese Academy of Sciences (CAS), Shenzhen, China; 2 Department of Microbiology, Stamford University Bangladesh, Dhaka, Bangladesh; 3 Department of Pathology, School of Basic Medicine, Henan University of Science and Technology, Luoyang, China; 4 Department of Microbiology, Jahangirnagar University, Savar, Bangladesh; 5 Laboratory of Environment Correlative Dietology, College of Food Science and Technology, Huazhong Agricultural University, Wuhan, Hubei, China; 6 Faculty of Pharmaceutical Sciences, Shenzhen University of Advanced Technology (SUAT), Shenzhen, China

**Keywords:** antimicrobial resistance, combination therapy, nanotherapy, phage delivery, phage engineering, precision medicine

## Abstract

The rising concern of antimicrobial resistance, coupled with the continually challenging management of complicated diseases such as cancer, has provided momentum toward precision molecular medicine. This review provides an overview of bacteriophage enabled strategies encompassing both conventional antibacterial applications and advanced bioengineered delivery systems. Recent advances in phage therapy include the use of tailored phage formulations, phage immobilization approaches and phage antibiotic combinations to achieve targeted bacterial lysis particularly against multidrug-resistant pathogens and biofilm-associated infections. Beyond their intrinsic antibacterial activity, phages can be genetically and chemically engineered as nanoscale scaffolds. Phage display technologies enable the incorporation of targeting ligands for selective binding to specific tissues including tumor cells. Furthermore, phage capsids can be modified to encapsulate and deliver diverse therapeutic payloads such as small-molecule drugs, nucleic acids and gene-editing systems such as CRISPR–Cas, thereby expanding their utility beyond infectious diseases. The integration of phage biology with nanobiotechnology positions these viral platforms at the forefront of next generation therapeutics. Engineered phages have demonstrated potential as precision delivery vectors for cytotoxic agents, immunomodulators and genetic material with improved specificity and reduced off-target effects. Emerging strategies including phage antibiotic conjugates and enzyme functionalized phages further enhance therapeutic efficacy and facilitate penetration of physiological barriers. Collectively, phage-based platforms represent a versatile and transformative approach with significant implications for the treatment of infectious, oncologic and genetic disorders, supporting the advancement of targeted and personalized medicine.

## 1 Introduction

The global rise in antimicrobial resistance has renewed interest in bacteriophages natural bacterial predators discovered over a century ago as alternative therapeutics. For clinical application, phages should be strictly lytic, highly efficient in bacterial killing, and thoroughly characterized to ensure safety. Although phage therapy shows strong potential against multidrug resistant infections, its use is often limited by the narrow host range of individual phages, necessitating personalized approaches. Phage cocktails targeting multiple bacterial receptors may broaden coverage and growing evidence supports their effectiveness against antibiotic resistant isolates (Kim et al., 2024). Cancer and genetic diseases represent two of the most formidable challenges in modern medicine, both rooted in molecular dysfunction whether in aberrant signaling, genetic mutation, or immune evasion (Mitra et al., 2024). Precision molecular techniques that target disease-specific biological markers have replaced nonspecific cytotoxic treatments as the mainstay of therapeutic methods during the last 20 years. Despite their effectiveness, conventional treatments like radiation and chemotherapy lack selectivity and are linked to systemic damage and resistance. By selectively disrupting carcinogenic pathways or modulating immunological checkpoints, targeted therapies such as monoclonal antibodies, small-molecule inhibitors, antibody-drug conjugates and immunotherapies increase efficacy while decreasing off-target consequences (Zafar et al., 2025). For instance, monoclonal antibodies like trastuzumab and cetuximab exemplify the precision of receptor blockade, while kinase inhibitors such as ponatinib highlight both the therapeutic power and safety challenges inherent to these strategies. Collectively, these agents have established a foundation for mechanism guided cancer therapy that bridges molecular pathology and clinical outcome (Shyam Sunder et al., 2023).

Conventional medicines are challenged by the growing burden of infectious diseases, genetic abnormalities and cancer. Even if classical vaccinations, radiation, chemotherapy and viral vectors have shown better results, their clinical utility is frequently constrained by manufacturing limitations, systemic toxicity, low targeted specificity and immune-related side effects (Vatsavai et al., 2025). These limitations have intensified interest in alternative delivery platforms that are both biologically safe and technologically adaptable. In recent years, bacteriophages viruses that naturally infect bacteria have emerged as promising nanocarriers capable of addressing several of these challenges across cancer therapy, gene delivery, and vaccine development (Wang et al., 2024). While conventional therapies face toxicity and targeting limitations, bacteriophage-based nanocarriers, plant virus nanoparticles (VNPs) and plant virus–like particles (VLPs) have emerged as promising alternatives, with phages enabling versatile delivery and plant viruses offering strong biocompatibility and structural uniformity (Caparco et al., 2024).

Compared to traditional viral vectors like lentiviruses and adenoviruses, bacteriophages have a number of benefits. They are inexpensive to make in large quantities, have little inherent toxicity and do not multiply in mammalian cells. Their sturdy capsids make them versatile platforms in immunotherapy, synthetic biology and nanotechnology by enabling precise genetic and chemical alterations that allow targeted ligands, antigens, or therapeutic proteins to be displayed on their surface (Karimi et al., 2016; González-Mora et al., 2020).

In oncology, phage display technology has enabled the identification and presentation of tumor-homing peptides that selectively recognize receptors overexpressed on cancer cells or tumor-associated vasculature, such as integrins and epidermal growth factor receptors. Filamentous phages like M13 have been widely explored for targeted delivery of chemotherapeutic agents, imaging probes, and phototherapeutic compounds, leading to enhanced tumor accumulation and reduced off-target toxicity (Bortot et al., 2022). Hybrid vectors, including adeno-associated virus/phage systems, further extend this approach by enabling receptor-mediated gene transfer and localized expression of suicide genes or immunomodulatory factors within tumor tissues (Pranjol and Hajitou, 2015). Bacteriophages have potential as platforms for delivering genes and proteins outside of oncology. While surface display systems offer controlled protein distribution through ligand-receptor interactions, their capacity to encapsulate nucleic acids permits the transport of therapeutic genes and genome-editing tools. These characteristics enable tissue-specific, regulated actions with less systemic harm (Rao and Zhu, 2022).

Phage-based vaccine delivery represents another rapidly advancing application. Unlike traditional vaccine platforms, bacteriophages offer exceptional thermal stability, repetitive antigen presentation, and intrinsic immunostimulatory activity. Phage display and phage DNA vaccine strategies have shown strong humoral and cellular immune responses in preclinical models, including studies targeting hepatitis C virus and SARS-CoV-2 (Davenport et al., 2022; Hasani et al., 2024). Notably, phage platforms have enabled rapid vaccine design, multivalent antigen presentation, and needle-free mucosal delivery approaches, highlighting their adaptability during pandemic situations (Zhu et al., 2024). Although challenges related to immune clearance, regulatory approval, and clinical translation remain, continued advances in phage engineering and delivery strategies are steadily overcoming these barriers. Collectively, bacteriophages represent a versatile and scalable platform with significant potential to reshape targeted cancer therapy, gene delivery, and next-generation vaccine development (Cui et al., 2024; Wang et al., 2024).

Phage-encoded proteins can act as small but potent blockers of key bacterial processes, halting a cell's motion. The ΦX174 protein E precisely disrupts cell walls by striking the membrane enzyme MraY, halting peptidoglycan formation (Orta et al., 2023). To wipe out competitors in complex microbial communities, many bacteria have borrowed bits of phages tailocins, for instance, tiny spear-like weapons made from phage tails that still strike with viral precision (Islam et al., 2019a; Jyotirmayee et al., 2024). These viral shifts highlight the molecular blueprints behind designing precise antimicrobials that barely touch unintended targets, revealing how a phage's structure constantly adapts to outsmart a bacterium's defenses (Shen and Loessner, 2021). Bacteria and their viruses keep sparring, each shifting tactics like rivals testing moves across a fogged Petri dish. In *Vibrio cholerae*, SXT integrative and conjugative elements (ICEs) not only spread antibiotic resistance but also arm the bacterium with defenses against phages. These elements aren't fixed they shift over time, and those changes can alter how easily phages slip into bacteria during an outbreak (Sarkar et al., 2019). This shows that resistance to antibiotics and phages often rises side by side, molded by the same forces like repeated exposure to harsh antimicrobial agents (Zhang and Ahn, 2025). Recent breakthroughs in medicine blend molecular engineering with the quiet genius of natural evolution, like designing proteins that fold with effortless precision. Modified phages can broaden their host range or pass along CRISPR-Cas modules to shut down resistance genes, while carefully designed phage blends block the bacteria's ways out (Dunne et al., 2021). Still, breaking down biofilms tough bacterial clusters that protect germs from antibiotics and the body's defenses is just as crucial for keeping infections under control. In infections caused by *Pseudomonas aeruginosa* and *Clostridium difficile*, evolution-trained phage cocktails and phage made enzymes such as M23 peptidases and depolymerases can tear through the sticky biofilm matrix, making the bacteria once again vulnerable to antibiotics (Dunne et al., 2021).

Bacteriophage based combination therapy refers to the effective use of phages combined with another antimicrobial approach to generate synergistic pathogen killing and overcome the limitations of single-agent phage treatment (Wang et al., 2024; Islam et al., 2025). Biofilms are also a barrier because biofilm associated bacteria can resist phage penetration and inhibit phage attachment (Park et al., 2024). Phages also struggle with multi-drug resistant (MDR) pathogens in adverse physiological conditions, and phage monotherapy may not always result in adequate bacterial clearance (Islam et al., 2019b; Soliman et al., 2023). Combination therapy is required to increase treatment efficacy, prevent resistance, and effectively break down the biofilms (Mayorga-Ramos et al., 2024). Several types of combination therapy are currently being developed. Phage-silver nanoparticle conjugate therapy combines phages and AgNPs. It makes use of the potent antibacterial properties of nano silver and the targeted specificity of phages. It facilitates the removal of biofilms and drug-resistant bacteria (Porter et al., 2021). Phage enzyme therapies involve use of phage-derived lytic enzymes to destroy bacterial cell walls or exopolysaccharide layers, which enables immediate cell lysis (Islam et al., 2020a,b). Phage-derived enzymes can enter bacteria inside dense biofilm matrices (Islam et al., 2021; Eghbalpoor et al., 2024). CRISPR-Cas9 mediated phage therapy involves generating phages to carry CRISPR-Cas9 systems that remove vital bacterial genes or resistance determinants, thereby including programmed death (Zhang et al., 2022). Overall, combination strategies represent a significant advancement in conventional phage therapy enhancing tissue penetration, improving selective bactericidal activity and increasing specificity against complex and multidrug-resistant bacterial infections (Zhang et al., 2023). The molecular principles of phage–bacteria interactions and the creation of engineered phages and phage-derived proteins are highlighted in this review, which combines recent developments in phage biology and therapeutic engineering. It also looks at new combination strategies including phage-encoded enzymes, CRISPR-enhanced phages and phage-nanoparticle systems. We also go over how advancements in phage technology, gene therapy, immunotherapy and targeted delivery platforms combine to influence next-generation antimicrobial tactics. We conclude by discussing the main translational issues and potential applications of phage-based therapies in precision medicine.

## 2 Targeted cancer therapy using bacteriophage

Cancer, which is characterized by uncontrolled proliferation of cells, invasion, metastasis, and genetic instability, is still one of the principal causes of illness and death worldwide. Conventional therapies such as chemotherapy and radiotherapy often suffer from limited specificity, systemic toxicity, and poor penetration into tumor tissues (Oluwajembola et al., 2025). These drawbacks underscore the urgent need for more selective, efficient, and safer delivery systems for anticancer agents. In recent years, bacteriophages viruses that naturally infect bacteria are gaining momentum as versatile nanocarriers for targeted cancer therapy, offering promising alternatives to classical chemotherapy and radiotherapy (Wang et al., 2024).

Phage-based targeted cancer therapy can be broadly classified into four major types, with ligand-mediated targeted drug delivery emerging as one of the most extensively studied and promising strategies, as it relies on highly specific molecular recognition to preferentially direct therapeutic agents to cancer cells or tumor vasculature, thereby enhancing efficacy while minimizing systemic toxicity (Węgrzyn et al., 2025). In this approach, bacteriophages particularly filamentous phages such as *M13* are genetically engineered via phage display technology to present tumor-specific peptides or ligands on their capsid proteins; notably, well-characterized motifs such as RGD peptides or EGFR-binding peptides enable selective recognition of receptors overexpressed on tumor cells or angiogenic endothelial cells (Foglizzo and Marchiò, 2021). Consequently, key molecular targets including integrins (e.g., αvβ3), epidermal growth factor receptor (EGFR), and other growth factor receptors associated with tumor progression and neovascularization can be efficiently exploited for selective tumor targeting. As a result, tumor-homing peptides identified through phage display facilitate the accumulation of drug-loaded phages at tumor sites, where they deliver chemotherapeutic agents, cytotoxic compounds, nanoparticles, or imaging agents directly to malignant tissues (Quilumba-Dutan et al., 2025). Importantly, this targeted localization markedly improves the therapeutic index by maximizing drug concentration at the tumor while reducing off-target effects in healthy tissues, while the modular nature of phage display further enables rapid adaptation of targeting ligands to diverse tumor markers, supporting personalized and precision oncology (Emencheta et al., 2024).

Photodynamic and photothermal phage-based cancer therapy represents the second major category of targeted phage therapeutics, in which bacteriophages act as nanoscale carriers for photosensitizers or photothermal agents. In this approach, phages such as M13 are engineered to display tumor-targeting peptides (e.g., EGFR-binding ligands), enabling selective accumulation on cancer cells (Venkataraman et al., 2025). Upon irradiation with light of an appropriate wavelength, the conjugated agents generate reactive oxygen species or localized heat, resulting in targeted tumor cell destruction. Experimental studies have demonstrated that EGFR-targeted M13 phages conjugated with photosensitizers can induce strong cytotoxic effects in ovarian and breast cancer models at very low doses, reflecting high targeting specificity and therapeutic precision. This strategy offers key advantages, including spatial and temporal control of therapy, minimal systemic toxicity, and non-invasive activation (Ulfo et al., 2022; Venkataraman et al., 2024).

The third category of phage-based cancer therapy is suicide-gene and pro-apoptotic protein therapy, which relies on engineered phages to deliver genes encoding toxic enzymes, tumor suppressor proteins (such as p53), or pro-apoptotic cytokines (e.g., TNF-α) that selectively trigger cancer cell death. In this approach, tumor specificity is achieved through ligand-mediated targeting and/or the use of cancer-specific promoters, ensuring localized gene expression within malignant tissues (Petrov et al., 2022). A prominent example is suicide-gene therapy using the AAVP (adeno-associated virus/phage) system, which integrates targeted delivery with intracellular prodrug activation. Here, an AAV genome encoding a suicide gene such as HSV-thymidine kinase (HSV-tk) is packaged within an M13 phage capsid displaying tumor-homing ligands like RGD4C, enabling selective binding to αvβ3 integrins expressed on tumor endothelium. Following gene transfer, systemic administration of a non-toxic prodrug results in its conversion into a cytotoxic metabolite specifically within tumor cells, producing efficient tumor killing along with a beneficial bystander effect (Staquicini et al., 2020). Overall, this strategy offers high tumor selectivity and minimal damage to normal tissues; however, its effectiveness depends on efficient gene expression, requires precise prodrug administration, and may be limited by host immune responses (Petrov et al., 2022).

The fourth category of phage-based targeted cancer therapy emphasizes tumor microenvironment immunomodulation and theranostic applications, in which bacteriophages are engineered to deliver immune-stimulatory molecules, tumor antigens, or immunomodulatory agents that reshape the tumor microenvironment and enhance anti-tumor immune responses. Through this approach, phage-based immunotherapies can promote immune cell infiltration, improve antigen presentation, and induce durable immune memory, thereby supporting long-term tumor control. In parallel, combination therapy and theranostic strategies exploit the structural versatility of phage capsids to co-deliver therapeutic payloads, such as drugs or genes, alongside diagnostic imaging agents including fluorescent dyes or contrast probes (Ooi and Yeh, 2024; Easwaran et al., 2025). Crucially, targeting is accomplished by ligands that identify microenvironment-associated indicators or tumor-specific receptors, enabling individualized, multimodal cancer treatment and real-time drug distribution and therapeutic response monitoring. Collectively, these approaches highlight the multifunctional potential of bacteriophages as precision oncology platforms with enhanced selectivity and reduced adverse effects compared to conventional therapies (Easwaran et al., 2025).

Overall, phage-based cancer treatments have a number of common drawbacks, such as immune clearance, restricted solid tumor penetration, and inconsistent payload loading or gene expression effectiveness. Therapy-specific requirements, like light activation in phototherapies or prodrug injection in suicide-gene methods, present additional difficulties. Clinical translation is also still hampered by formulation complexity, large-scale production, and regulatory obstacles. To progress phage-based platforms toward efficient and broadly applicable cancer treatments, these problems must be resolved (Cui et al., 2024). However, several types of bacteriophages have been engineered for targeted cancer therapy, emphasizing their potential as precision delivery platforms (Table 1).

**Table 1:** Bacteriophage types utilized in targeted cancer therapy.

Phage type	Representative phage	Targeting ligand/target	Targeted cancer therapy application	Key advantage in targeting	References
Filamentous phage (native display)	M13, fd	RGD peptides → αvβ3/αvβ5 integrins	Targeted delivery of chemotherapeutic drugs or imaging agents to tumor vasculature	High-density surface display of targeting peptides; strong tumor-homing ability	Kim et al., 2011
Engineered filamentous phage	M13-based targeted phage	EGFR-binding peptides	Targeted photodynamic or photothermal therapy in EGFR-overexpressing tumors	Precise receptor-specific targeting with external activation control	Zhang et al., 2019
AAV/phage hybrid vector (AAVP)	M13 capsid + AAV genome	RGD4C peptide → αvβ3 integrin	Targeted suicide-gene therapy (HSV-tk), cytokine gene delivery	Combines phage targeting with efficient mammalian gene expression	Biswas et al., 2019
Phage-based gene delivery vector	T7, λ (Lambda)	Tumor-homing peptides or antibodies	Targeted gene delivery and experimental CRISPR/Cas9-based gene editing	Large DNA packaging capacity; genetic stability	Bastard et al., 2021
Phage nanoparticle scaffold	M13-derived nanoparticles	Tumor-specific peptides (e.g., integrin-, EGFR-targeting)	Targeted delivery of cytokines or immune-modulatory proteins to tumor microenvironment	Direct localization of therapeutic proteins at tumor site	Montaut et al., 2018
Phage-based cancer vaccine (targeted)	T4, M13	Tumor-associated antigens (TAAs)	Targeted induction of anti-tumor immune responses	Intrinsic adjuvant effect with antigen-specific targeting	Bastard et al., 2021
Multifunctional targeted phage (theranostics)	M13 multifunctional phage	Tumor receptors + imaging probes	Targeted therapy combined with diagnostic imaging	Simultaneous tumor targeting, treatment, and monitoring	Montaut et al., 2018

## 3 Gene and protein therapy using bacteriophage

Phages have a simpler structure, are comparatively non-pathogenic to human cells, and may be precisely genetically modified to carry particular therapeutic cargo, in contrast to traditional viral vectors like adenovirus or lentivirus (Sittiju et al., 2024). The fundamental rationale for using phages in gene therapy is their natural ability to wrap DNA and transmit it into target cells. The phage capsid protects its genetic material in conventional phage infection cycles before injecting it into a bacterial host and utilizing the host's machinery to proliferate (Petrov et al., 2022; Wang et al., 2024). This efficient packaging technique has been utilized to produce phage vectors that, when built correctly, carry therapeutic genes rather than bacterial replication genes, enabling receptor-mediated gene transfer into mammalian cells. One important development is the engineering of filamentous phage particles, such as M13, to increase the effectiveness of gene delivery. In order to improve cell transduction efficiency while guaranteeing specificity toward cells overexpressing a particular antigen, researchers recently created what they named a “TransPhage,” in which the phage genome was reduced. A dual gene-mediated therapeutic effect was demonstrated in cancer models using TransPhage carrying a gene for a membrane-bound Fc fragment, which increased tumor cell death through immune cell engagement (Kao et al., 2023).

In the context of cancer gene therapy, phage vectors have shown promise not only for delivering single therapeutic genes but also for complex gene editing systems such as CRISPR/Cas9. For example, hybrid vectors combining phage components with adeno-associated viral elements have been used to deliver CRISPR/Cas9 components targeting mutant p53 genes in lung cancer cells. This approach restores functional tumor suppressor activity and highlights the potential of phage systems for precise genomic editing therapies (Yang Zhou et al., 2020; Petrov et al., 2022).

The low immunogenicity and modular engineering profiles of phages make them attractive for protein therapy as well. Notably, phage display technology enables peptides or proteins to be presented on the phage surface, facilitating targeted distribution to specific tissues or organs through ligand–receptor interactions. In oncological applications, phage particles have been engineered to simultaneously display therapeutic proteins and tumor-targeting ligands, allowing modulation of cellular signaling pathways or immune responses in a highly localized manner (Sharifi et al., 2024). Beyond cancer, phage-mediated gene and protein delivery has shown promise in other disease contexts, particularly inflammatory disorders. For example, engineered phages have been explored as anti-inflammatory protein delivery systems in gastrointestinal disease models, where phages were designed to educate gut bacteria to produce therapeutic peptides *in situ*. Importantly, preclinical studies demonstrated that such approaches significantly reduced inflammation in disease-model mice, underscoring the versatility of phage platforms for localized protein production and targeted therapy (Islam et al., 2024b). From a molecular engineering perspective, phages offer exceptional flexibility, as genetic modifications can incorporate regulatory elements for controlled gene expression, tissue-specific targeting peptides, and immunomodulatory proteins that recruit or activate host immune cells at disease sites. Crucially, these engineered features can be integrated within a single phage particle, enabling sophisticated multimodal therapeutic strategies that extend well beyond conventional gene replacement approaches (Wang et al., 2024; Venkataraman et al., 2025). Overall, bacteriophage-based gene and protein therapy represents a novel and promising frontier in biotechnology, merging the natural efficiency of phage genetics with modern engineering to deliver targeted therapeutic agents. Continued innovation in vector design, cargo engineering, and delivery strategies is poised to expand the utility of phages in treating a broad spectrum of diseases, from resistant bacterial infections to cancer and beyond (Cui et al., 2024; Sahoo et al., 2024).

However, a summary of major phage-enabled delivery platforms, their therapeutic cargo, biomedical applications, and key advantages is presented in Table 2.

**Table 2:** Phage and viral nanoplatforms for targeted cancer drug delivery and gene therapy.

Delivery platform	Cargo type	Application	Advantages	References
Filamentous phage (M13)	Drugs, peptides, nanoparticles	Targeted cancer therapy	High targeting specificity through phage display peptides; easy genetic modification	Peiseler and Tacke, 2021
AAVP hybrid phage vector	Suicide genes (e.g., HSV-tk), therapeutic DNA	Cancer gene therapy	Targeted gene delivery with receptor-mediated binding and prodrug activation	Petrov et al., 2022
Phage–photosensitizer conjugates (M13-based)	Photosensitizers, photothermal agents	Photodynamic/photothermal cancer therapy	Spatial and temporal control of therapy; minimal systemic toxicity	Cai et al., 2025
Phage nanoparticles (general bacteriophages)	Chemotherapeutic drugs, imaging agents	Cancer targeting and theranostics	Tumor-specific targeting and combined therapy + diagnostics	Emencheta et al., 2024
Plant virus nanoparticles (VNPs)/virus-like particles (VLPs)	Antigens, immunostimulatory molecules, drugs	Cancer immunotherapy	High biosafety, strong immune activation, easy surface modification	Ooi and Yeh, 2024
Engineered phage vectors (TransPhage)	Therapeutic genes, immune-activating proteins	Cancer gene therapy	Efficient gene delivery and immune cell recruitment	Petrov et al., 2022
Phage-CRISPR hybrid vectors	CRISPR/Cas9 gene editing system	Precision cancer gene editing	Precise genome targeting and restoration of tumor suppressor function	Rauf et al., 2025

## 4 Vaccine delivery via bacteriophage

The persistent increase in infectious diseases and the drawbacks of conventional vaccination platforms have spurred the search for novel, secure, and adaptable vaccine delivery systems. In this sense, bacteriophages viruses that specifically infect bacteria have attracted attention as possible vaccine carriers. Their unique capsid geometries allow for precise genetic and chemical modifications, and their inability to infect mammalian cells makes them intrinsically safe for human use (González-Mora et al., 2020). Unlike protein subunit vaccines and attenuated viral vaccines, which have the risk of reverting to virulence, bacteriophages can supply antigens in highly ordered, repetitive arrays that successfully increase immune recognition. Because of these features, bacteriophages serve as a unique bridge connecting immunology, synthetic biology, and nanotechnology in the creation of modern vaccines (Hess and Jewell, 2020).

**Figure 1:**
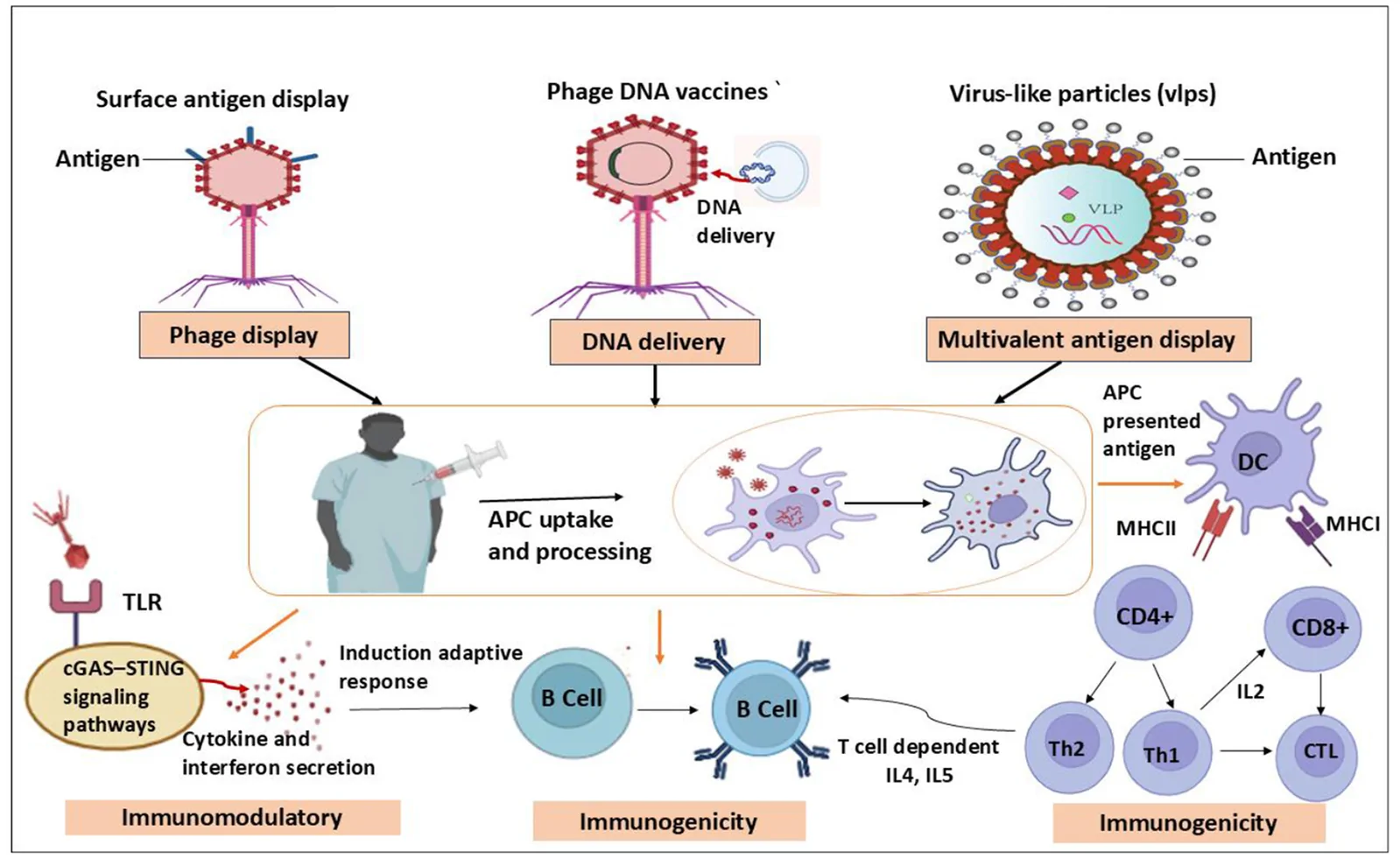
Bacteriophage-based vaccine delivery platforms. The figure illustrates three major bacteriophage-based vaccine delivery strategies: phage surface antigen display, phage DNA vaccines, and virus-like particles (VLPs) for antigen presentation. In these systems, antigens or DNA cargos are loaded onto or within phage particles, enabling efficient delivery to antigen-presenting cells (APCs) after administration. Following uptake, APCs process the antigens and present them through MHC class I and II pathways, activating innate immune signaling pathways such as TLR and cGAS–STING, which promote cytokine and interferon secretion. This process subsequently stimulates adaptive immune responses, including activation of B cells, CD4^+^ helper T cells, and CD8^+^ cytotoxic T cells, leading to enhanced immunogenicity and protective immunity.

Bacteriophage-based vaccines take advantage of the molecular principles of phage display, a method that presents foreign antigenic peptides or proteins on the surface by genetically fusing them to phage coat proteins. By imitating the structural patterns present on numerous infections, this repeating epitope presentation increases immune system activation. Phage particles are easily absorbed by antigen-presenting cells, such as macrophages and dendritic cells, after which the antigens are processed and presented via major histocompatibility complex pathways. Strong humoral and cellular immune responses are produced as a result of this mechanism, which activates both T and B cells (Figure 1; González-Mora et al., 2020). Crucially, by activating innate immune receptors, bacteriophages themselves function as natural adjuvants, lowering or doing away with the requirement for other adjuvant substances. In addition to presenting surface antigens, bacteriophages have been developed as carriers of DNA vaccinations (Bao et al., 2019). In these systems, the modified phage genomes contain eukaryotic expression cassettes that express target antigens. When phage particles are consumed by host cells, the encoded antigen genes can be generated, mimicking the action of conventional DNA vaccines while utilizing the structural protection and efficient delivery of the phage capsid. Preclinical studies have shown that this strategy addresses the main problems of naked DNA vaccines, such as poor cellular uptake and rapid degradation, while simultaneously enhancing antigen expression and immune activation (Xu et al., 2018).

Numerous practical research examples have shown the great potential of bacteriophage-based vaccine delivery. The versatility and synergistic potential of phage platforms were demonstrated in hepatitis C virus studies, where lambda bacteriophages modified to express the HCV core antigen produced stronger immune responses than traditional plasmid DNA vaccines in animal models, especially when used in heterologous prime–boost regimens (Saeedi et al., 2014; Zalewska-Piatek and Piatek, 2021). Notably, phage-based vaccines attracted attention during the COVID-19 pandemic due to their quick scalability and versatility. Phage display systems that target the SARS-CoV-2 spike protein produced strong antibody responses in animals and allowed for novel needle-free delivery techniques like intranasal and aerosolized administration. Moreover, advanced platforms such as bacteriophage T4 significantly expanded vaccine design by enabling multivalent antigen display, which produced strong systemic and mucosal immune responses and protection against lethal virus challenge in mouse models (Zhu et al., 2022; Ul Haq et al., 2023). Additionally, bacteriophage-derived virus-like particles, particularly AP205 VLPs (Virus-Like Particles), have shown the ability to produce potent neutralizing antibody responses against SARS-CoV-2 while maintaining superior safety profiles, demonstrating that phage-based vaccine technologies can rival established vaccine platforms in terms of both immunogenicity and adaptability (Liu et al., 2021).

Finally, although there are still challenges with quality assurance, immunological clearance, and regulatory approval, bacteriophage-based vaccines have many benefits, including as inexpensive production costs, high thermal stability, genetic diversity, and inherent adjuvant activity (Aghebati-Maleki et al., 2016). Phage-mediated vaccine platforms are positioned as promising, scalable, and adaptable tools for next-generation vaccination strategies in both infectious disease prevention and therapeutic immunization, especially in resource-limited settings, thanks to ongoing advancements in synthetic biology and immunoengineering (Ul Haq et al., 2023).

## 5 Mechanism of the ΦX174 phage-encoded protein antibiotic

Bacteriophage ΦX14's protein E is a minimalist protein antibiotic that prevents the production of bacterial cell walls by blocking the vital membrane enzyme MraY, which is responsible for the generation of lipid I. The E–MraY–SlyD (YES) complex was resolved by cryo-EM investigations, which demonstrated how E inserts its N-terminal transmembrane helix into MraY's catalytic groove. Peptidoglycan construction is stopped when a conserved proline causes a bend in the helix, sterically preventing substrate access (Orta et al., 2023). This process depends on the host chaperone SlyD, which stabilizes protein E during membrane insertion and promotes effective complex formation *in vivo* (Orta et al., 2023). Furthermore, structural study showed that E binding recruits ordered lipid molecules and causes conformational rearrangements in MraY's transmembrane helices, highlighting the crucial role that membrane topology plays in the inhibitory process (Dash et al., 2025). Triazinedione-based peptidomimetics that imitate the inhibitory motif of E were developed to illustrate the translational importance of this interface (Fairbairn and Rafikov, 2025). These substances established the E–MraY interaction as a viable antibacterial target by effectively inhibiting MraY and working in concert with bacitracin to combat multidrug-resistant infections (Dash et al., 2025).

## 6 Competitive suppression by a phage tail like bacteriocin

An example of how pathogenic bacteria adapt viral components to stifle competition in wild populations is offered by phage tail like bacteriocins, also known as tailocins (Scholl, 2017; Zhang et al., 2023). *Pseudomonas* strains preferentially use tailocins, which are structurally similar to bacteriophage tails and encoded inside genomic loci derived from prophages to eradicate closely related competitors (Backman et al., 2024). In order to provide great specificity in interstrain competition, each strain develops a unique tailocin variation that targets particular lipopolysaccharide receptors on the surface of susceptible bacterial cells (Backman et al., 2024). The remarkable stability of both tailocin variants and their corresponding receptors was found through longitudinal analysis of historical herbarium samples spanning 170 years. This suggests that these elements are subject to intense selection and have co-evolved with their bacterial hosts over centuries (Backman et al., 2024). Tailocin mediated killing is mechanistically similar to phage infection: target receptors are recognized by tail fibers, and sheath contraction causes a fatal puncture in the rival cell membrane, which causes fast cell death (Backman et al., 2024). Beyond its ecological role, tailocins have a great deal of promise for translation. Because of their target specificity and diversity, they are excellent candidates for the creation of tailocin cocktails, which are intended to eradicate pathogenic strains while preserving beneficial microbiota (Backman et al., 2024). All of these results point to the adaptive inventiveness of bacteria in using viral machinery for their own gain, and they establish tailocins as a viable class of precision antimicrobial medicines (Shim, 2023).

## 7 Temporal resistance dynamics in phage pathogen conflict

The temporal dynamics of bacterial mobile genetic elements mediating resistance greatly influence phage pathogen interactions. Longitudinal investigations of *Vibrio cholerae* have shown that changes in SXT integrative and conjugative elements (ICEs), which concurrently confer resistance to both antibiotics and phages, are the primary cause of fluctuations in phage susceptibility (Grygiel et al., 2024). The co-dissemination of antibiotic and phage resistance traits is encouraged by the presence of defense systems in these ICEs at particular genetic hotspots and the ability of phage infection to initiate high-frequency ICE conjugation (Islam et al., 2023; Podlacha et al., 2024). A dynamic evolutionary arms race is highlighted by the ability of phages to respond to these bacterial defenses by developing new inhibitors that negate SXT-mediated protection (Legault et al., 2021). These insights have been further developed by recent research: phage predation exerts selective pressure that shapes pathogen genetic diversity and influences disease severity (Madi et al., 2024), while rapid turnover of mobile genetic elements has been demonstrated to accelerate adaptation and the spread of resistance within bacterial populations (Ghaly and Gillings, 2022). Together, these results highlight the intricate relationship between phages and bacterial resistance elements, highlighting the importance of temporal changes in resistance determinants in comprehending bacterial evolution and creating successful phage-based treatments (Oromí-Bosch et al., 2023).

## 8 Phage therapy: biology and prospects

Phage therapy is gaining ground as a hopeful alternative to traditional antibiotics, especially as antimicrobial resistance spreads worldwide, a stubborn problem that lingers despite decades of medical progress and the sharp scent of hospital disinfectant (Miethke et al., 2021). Bacteriophages are viruses that target only bacteria and kick off the lytic cycle by latching onto the cell's surface receptors, slipping their genetic material inside, and multiplying rapidly within the host (Parija, 2023). The process ends with the cell breaking open, spilling countless new phages into their surroundings and wiping out the bacterial population (Strathdee et al., 2023).

**Figure 2:**
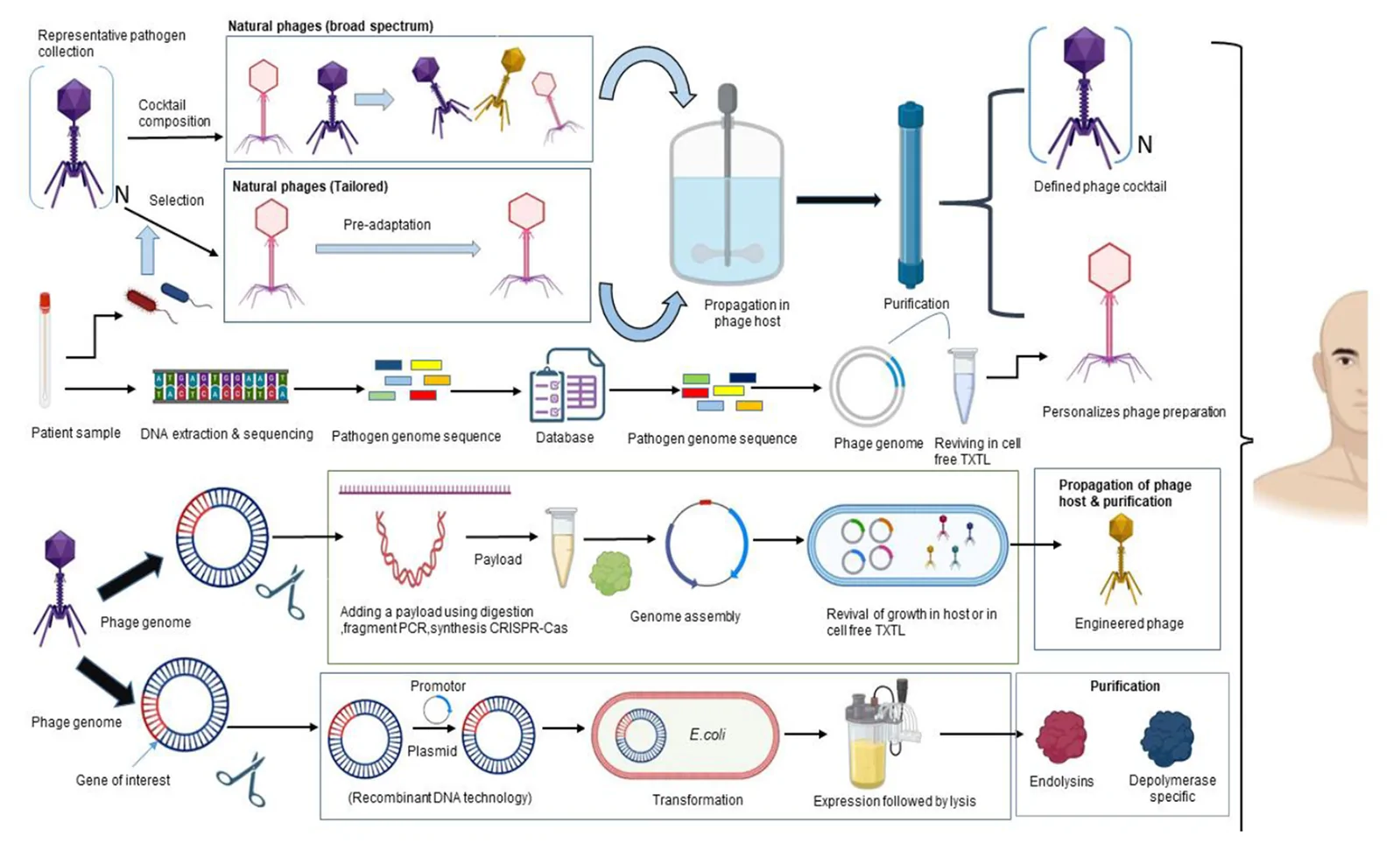
Synthetic phage engineering and rational cocktail design.

Phage therapy, unlike broad-spectrum antibiotics, works with remarkable precision zeroing in on harmful bacteria while leaving beneficial microbes untouched (Palma and Qi, 2024). Still, bacteria can evade phages by altering their receptors, using restriction–modification defenses, or deploying CRISPR-Cas immunity, so designing treatments demands careful thought (Al-Ouqaili et al., 2025). Recent studies show that phages and their bacterial hosts evolve side by side, with mobile genetic elements like SXT ICEs swapping in and out at a rapid pace changes that can shape how well phages work and how resistance traits spread (Hussain et al., 2021). Phage predators influence how pathogen populations grow and change shaping their diversity, virulence, and even the course of disease underscoring the ecological and evolutionary implications of phage therapy (Brives et al., 2024). Taken together, the findings highlight the urgency of clear clinical guidelines, tailored phage cocktail combinations, and steady tracking of how phages and bacteria interact like noting subtle shifts in culture growth to boost treatment results (Hussain et al., 2021; Strathdee et al., 2023). Two complementary approaches to long-lasting phage therapy are shown in Figure 2. In order to restrict bacterial escape and frequently promote evolution toward weaker, less fit resistance, the first employs combinations of various wild-type lytic phages. In the second, phage potency is increased through genetic engineering, either by expanding the host range or by introducing tools like CRISPR-Cas to destroy resistance genes. Molecular precision and evolutionary control work together to provide long-lasting therapeutic efficacy.

## 9 Evolving phage cocktails to target biofilm

Bacteriophage cocktails shaped through evolutionary training could be an effective strategy to break down *Pseudomonas aeruginosa* biofilms, whose dense, viscous extracellular polymeric matrix and antibiotic-tolerant persister cells make them difficult for standard antibiotics to penetrate (Urgeya et al., 2025). Repeatedly passing phages through bacteria adapted to biofilms expands their host range, increases replication, and enhances their ability to break down biofilms, producing phages that can target mixed bacterial pockets within the biofilm, thereby restoring antibiotic efficacy against multidrug-resistant strains (Kunisch et al., 2024). This approach takes advantage of bacterial resistance trade-offs: mutations that help bacteria fend off phages often make them more vulnerable to certain antibiotics, bringing back the drugs' effectiveness against stubborn multidrug-resistant strains (Kunisch et al., 2024). A two-phage cocktail, built from phages trained through evolutionary methods, sharply cut biofilm mass and bacterial numbers without sparking resistance to both, hinting that this kind of pairing could stay effective over time and keep new resistance from arising (Abedon et al., 2021). These evolved phages exploit multiple bacterial receptors for adsorption, thereby reducing the likelihood of resistance arising through single-receptor modification and increasing treatment success, and subsequently break down bacterial cells through complementary lytic mechanisms (Hasan and Ahn, 2022). Working in tandem, the findings show that evolution-guided phage therapy can break down tough biofilms and bolster standard antibiotics, a flexible strategy against stubborn P like a scalpel shaving away dense, gritty buildup (Lin et al., 2025). *P. aeruginosa* infections often strike in hospitals, clinging to tubing or lingering on damp surfaces (Islam et al., 2024a; Yetiş et al., 2024). Table 3 explains *Pseudomonas aeruginosa* that are biofilm-forming and multidrug resistant pose significant clinical concerns because of their innate resistance to traditional medicines. Strong planktonic growth suppression and biofilm reduction are demonstrated by the bacteriophages and phage cocktails listed here in a variety of clinical and laboratory strains. Significant biofilm biomass reduction was accomplished by Jumbo Myoviridae phages (φKZ, vB_PaerM_AttikonH10) and Podoviridae phages (DiSu5, φParuNE1), with certain phages showing synergistic effects when paired with antibiotics. Phage cocktails, such as DL52–DL62, allowed for >95% biofilm eradication demonstrating the advantage of broad host-range combinations. These findings, which are supported by both *in vitro* and *in vivo* data, demonstrate the translational potential of bacteriophage-based methods as stand-alone or supplemental therapy against resistant *P. aeruginosa* infections.

**Table 3:** Anti-biofilm activity of bacteriophages.

Phage	Bacterial strain	Phage family/genome	Biofilm/growth effect	Treatment type	Application potential	References
phiKZ	PA01	Myoviridae, jumbo dsDNA	Moderate biofilm suppression;	Phage alone; Phage + Antibiotic (Aztreonam-Lysine, sub-MIC)	Useful in phage–antibiotic synergy (PAS) strategies to combat *P. aeruginosa* biofilms	Davis et al., 2021
DiSu3	PA235	*Myoviridae*/dsDNA	83% biofilm inhibition	Phage alone	Strong anti-biofilm potential	Urgeya et al., 2025
DiSu3	PA235	*Myoviridae*/dsDNA	90% reduction of preformed biofilm	Phage alone	Rapid action	Urgeya et al., 2025
DiSu5	PA209	*Podoviridae*/dsDNA	80% bacterial growth inhibition	Phage alone	High killing efficiency	Urgeya et al., 2025
DiSu5	PA235	*Podoviridae*/dsDNA	96% growth inhibition	Phage alone	Strong lytic potential	Urgeya et al., 2025
ΦParuNE1	MDR *P. aeruginosa* clinical isolates	Podoviridae/dsDNA	>60% biofilm biomass removal	Phage alone	Promising for targeting MDR *P. aeruginosa*	Enwuru et al., 2021
vB_PaerM_AttikonH10	MBL-PA (Metallo-β-lactamase producing)	Chimalliviridae, Phikzvirus/jumbo dsDNA	>94% planktonic growth inhibition (*in vitro*) & *in vivo* efficacy	Phage alone; Phage + Antibiotic (Amikacin)	Broad host-range lytic phage with good PK, synergizes with amikacin *in vivo* promising for therapeutic use against MBL-PA infections	Paranos et al., 2025
Cocktail (DL52, DL60, DL62, etc.)	PAO1	Myoviridae (cocktail)	>95% reduction of biofilm	Phage cocktail	Very effective biofilm dispersal; good for therapeutic development	Alves et al., 2016
vB_PaN_phPA-Intesti	*P. aeruginosa* urinary	Lytic	5.5 log CFU/cm^2^ reduction of biofilm in urine	Phage alone; Phage + Antibiotic (Ciprofloxacin)	Very effective against urinary biofilms; potential for UTI phage therapy	Martinho et al., 2024

## 10 Engineering M23 peptidase for anti-*Listeria* activity

Antibiotic-resistant *Listeria monocytogenes* is on the rise, underscoring the need for new strategies to control this pathogen and prevent severe disease. The M23 family of peptidases, which are metalloenzymes by nature are gaining attention for their knack at breaking down peptidoglycan in bacterial cell walls, disrupting that rigid layer through targeted degradation of its structural components Recent studies found new M23 peptidases that can break down by efficiently cleaving its cell wall peptidoglycan. *L. monocytogenes* and other Gram-positive bacteria with meso-diaminopimelic acid–type peptidoglycan (Kaus-Drobek et al., 2025), their cell walls firm as dry parchment. For example, the M23 peptidase from *Streptococcus thermophilus* showed strong antibacterial action enough to drive the design of chimeric variants with sharper targeting and greater potency (Razew et al., 2022). By mapping the active site's shape and pinpointing how each enzyme selects its substrate, structural analyses have opened the door to designing sharper, more potent antimicrobial agents (Zhou and Huang, 2024). These engineered M23 peptidases have already shown real promise for food safety, knocking out before it can spread like removing a sour note before it spoils the whole batch. Monocytogenes picked up from contaminated surfaces show promise for both industrial and clinical uses, from factory floors to hospital equipment (Kaus-Drobek et al., 2025). Taken together, the results highlight how M23 peptidases could become powerful next-generation antimicrobials, linking core enzymology to real-world microbiology and tackling persistent Listeria infections that form surface-associated biofilms (Kaus-Drobek et al., 2025).

## 11 Phage depolymerase mediated antibiofilm activity

Researchers often rely on microtiter biofilm assays to gauge how well therapeutic agents work against biofilms, from bacteriophages to their depolymerases, typically assessing changes in biomass or optical measurements as indicators of activity researchers studying *Klebsiella* phage KP34 and its recombinant depolymerase KP34p57 have used familiar microtiter-based techniques, colony counts, LIVE/DEAD BacLight staining, and crystal violet biofilm assays to measure how effectively these agents inhibit and break down biofilms, noting changes from dense, opaque layers to clearer wells (Zurabov et al., 2023; Jiao et al., 2025). The colony count method tracks bacteria that can grow, giving clear proof of their survival or showing when their growth's been stopped like spotting only a few pale colonies on a petri dish. The LIVE/DEAD BacLight assay separates living cells from dead ones, revealing whether a treatment kills bacteria outright or simply stops them from multiplying like spotting bright green survivors amid a scatter of dull red corpses. In contrast, CV staining gauges the total biofilm biomass by tracking how much dye it holds, a sign of its density and structural strength, much like color settling deep into fabric (Wang and Wu, 2024). Still, every one of these assays comes with its own built in limits like a scale that never measures quite the same twice. Colony counts can miss signs of antibiofilm activity, since some bacteria tucked inside the biofilm remain alive but won't grow in culture (Latka et al., 2021; Islam, 2025). The LIVE/DEAD BacLight assay can give skewed results when depolymerases or similar agents can alter cell morphology and affect dye permeability, leading to false-positive viability signals (Lucidi et al., 2024). CV staining is a reliable way to gauge biofilm biomass, but it can mislead especially when depolymerases break down polysaccharides, freeing bits of the matrix that catch the dye and make the biofilm look heavier than it really is (Jiao et al., 2025). Even with their limits, microtiter biofilm assays still serve as a go-to for early screening of antibiofilm agents, quickly revealing hints of activity like a faint halo in a well plate. By combining complementary assays such as crisp, high resolution microscopy with precise molecular techniques you can gain a fuller, more accurate picture of antibiofilm activity (Latka et al., 2021).

## 12 Regulators of biofilm formation in *C. difficile*

*Clostridioides difficile*, a major culprit behind hospital-acquired antibiotic-related diarrhea, forms intricate biofilms composed of structured, surface-associated layers that promote its persistence and contribute to recurrent infection. Recent studies have shed light on how extracellular DNA, cell-surface proteins, and the secondary messenger cyclic-di-GMP work together to drive biofilm growth, from the sticky first layer to its dense, structured form. eDNA forms a key part of the biofilm's framework, holding it together like a mesh of fine threads and helping it stand up to harsh environmental pressures. They found that heavier biofilm growth tended to come with more frequent sporulation and a richer mix of eDNA, like a thick, cloudy layer clinging to the surface. Certain *difficile* lineages strains like 630 (RT012), CD305 (RT023), and M120 (RT078) carry noticeably more eDNA than others, including R20291 (RT027) and M68 (RT017; Xaplanteri et al., 2025). Cell surface proteins are crucial in biofilm formation, facilitating bacterial attachment to surfaces. Researchers have found four of these proteins two controlled by c-di-GMP (CD2831 and CD3246) and two that operate without it (CD3392 and CD0183). High c-di-GMP levels boost the production of CD2831 and CD3246, which sortase SrtB locks firmly into the cell wall like rivets in steel. These proteins help kickstart biofilm growth, and when they're overproduced, the biofilm swells into a thicker, denser layer (Ouyang et al., 2024). When c-di-GMP levels drop, the metalloprotease PPEP-1 slices these proteins off the cell wall, leaving fewer behind to help build the biofilm's sticky matrix. eDNA, cell surface proteins, and c-di-GMP work together in a finely tuned network that controls biofilm formation much like gears meshing in a well-oiled machine. It's tough, like trying to thread a needle in dim light. To break down biofilm integrity, we first need to understand how its mechanisms work imagine prying apart the sticky layers on a river rock (Vuotto et al., 2024).

## 13 Combination therapy

Bacteriophage based combination therapy refers to the effective use of phages combined with another antimicrobial approach to generate synergistic pathogen killing and overcome the limitations of single-agent phage treatment (Wang et al., 2024). Although lytic phage therapy has great specificity and the potential to disrupt bacterial populations in a targeted manner, many bacteria can survive and bypass the destruction by lytic phage (Palma and Qi, 2024). It can limit the effectiveness of phage therapy alone. Biofilms are also a barrier because biofilm-associated bacteria can resist phage penetration and inhibit phage attachment (Park et al., 2024). Phages also struggle with multi-drug resistant (MDR) pathogens in adverse physiological conditions, and phage monotherapy may not always result in adequate bacterial clearance (Soliman et al., 2023). Following the limitations of conventional antibacterial therapies, bacteriophages have emerged as highly specific agents capable of selectively infecting and lysing target bacteria while largely preserving the surrounding microbiota. However, phage therapy faces several challenges, including a narrow host range, limited biofilm penetration, and the potential for bacterial resistance. To overcome these limitations, engineered phage-based delivery systems have been developed that utilize natural phage infection mechanisms while incorporating genetic or chemical modifications to enhance antibacterial efficacy, targeting and stability. In addition, phages can be engineered to deliver antibacterial cargos such as enzymes or antimicrobial peptides. Complementary approaches, including CRISPR–Cas–based antibacterial systems and nanoparticle-assisted delivery platforms, are also being explored to enhance bacterial targeting and biofilm disruption. Together, these strategies represent an emerging generation of multifunctional phage therapeutics aimed at combating antimicrobial resistance.

**Figure 3:**
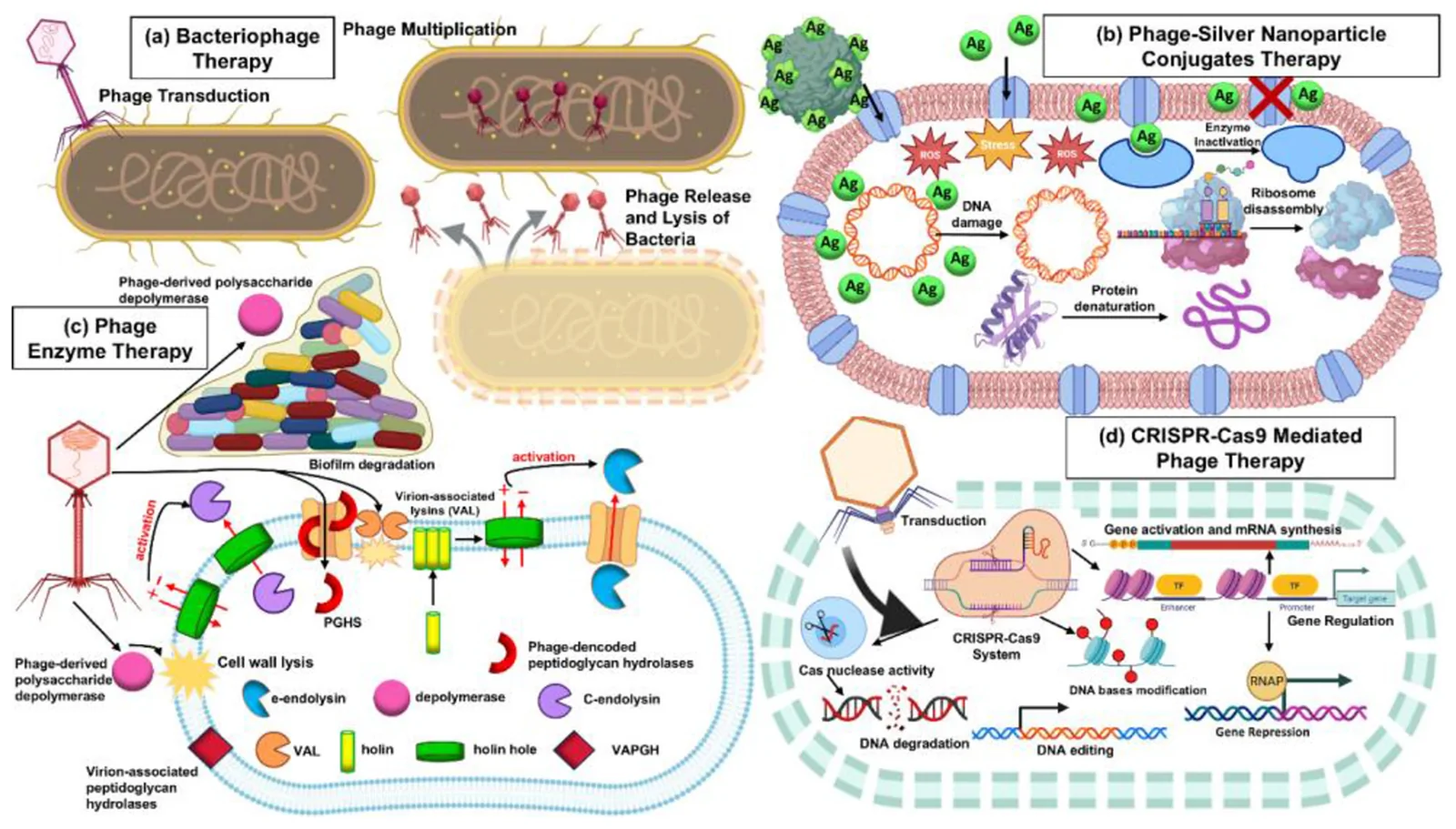
Combination therapies using bacteriophage-based interventions against bacterial pathogens. **(a)** Conventional bacteriophage therapy; utilizes lytic phages that selectively infect and lyse target bacteria. **(b)** Phage-silver nanoparticle conjugate therapy; integrates the host-specific binding ability of phages with the broad-spectrum antimicrobial activity of nanosilver to enhance bacterial killing. **(c)** Phage-derived enzyme therapy; employs purified phage lysins or depolymerases to degrade bacterial cell wall polysaccharides and biofilm matrices, enabling rapid cell lysis and biofilm disruption. **(d)** CRISPR-Cas9 mediated phage therapy; uses genetically engineered phages carrying CRISPR-Cas9 constructs designed to target and cleave essential bacterial genes or resistance determinants, enabling precise, programmable eradication of resistant strains.

Combination therapy is required to increase treatment efficacy, prevent resistance, and effectively break down the biofilms (Mayorga-Ramos et al., 2024). Several types of combination therapy are currently being developed. Phage-silver nanoparticle conjugate therapy combines phages and AgNPs. It makes use of the potent antibacterial properties of nano silver and the targeted specificity of phages. It facilitates the removal of biofilms and drug-resistant bacteria (Porter et al., 2021). Phage enzyme therapies involve use of phage-derived lytic enzymes to destroy bacterial cell walls or exopolysaccharide layers, which enables immediate cell lysis. Phage-derived enzymes can enter bacteria inside dense biofilm matrices (Eghbalpoor et al., 2024). CRISPR-Cas9 mediated phage therapy involves generating phages to carry CRISPR-Cas9 systems that remove vital bacterial genes or resistance determinants, which allows programmed death (Zhang et al., 2022). Overall, combination techniques appear to be an effective advancement in conventional phage therapy, capable of increasing the penetration ability, selective killing efficacy, and specificity against complicated and resistant bacterial infections (Table 4). The Figure 3a represents conventional bacteriophage therapy, in which virulent, host-specific phages adhere to bacterial receptors, inject their genetic material by transduction, and multiply to reproduce within the host cell. These phages cause cell lysis and release progeny phages that spread across the infection site. This method is very selective and can eliminate infections while preserving beneficial bacteria.

**Table 4:** Comparison of major phage combination therapy strategies.

Combination strategy	Mechanism of synergy	Target applications	Key advantages	Challenges	References
Phage-antibiotics	Antibiotics weaken cell wall, improving phage penetration; phage disrupt biofilm to enhance antibiotic diffusion; phage-driven resensitization to antibiotics	MDR infections, chronic biofilms, *P. aeruginosa, S. aureus, A. baumannii*	Strong synergistic killing; reduces antibiotic dosage; effective against biofilms and persisters	Timing/dosage critical; risk of antagonism; may not work for all strains	Grygiel et al., 2024; Nawaz et al., 2024
Phage-silver nanoparticles (AgNPs)	AgNPs disrupt membrane integrity → greater phage adsorption; phage guides AgNPs to target cells; combined oxidative + lytic killing	Biofilm infections, wound pathogens, *E. coli, S. aureus, P. aeruginosa*	Potent dual-acting system; effective at low NP concentration; eco-friendly when using green AgNPs; reduces silver toxicity	Potential nanoparticle toxicity; AgNP resistance (e.g., *A. baumannii*); stability issues; regulatory challenges	De Plano et al., 2025; Wdowiak et al., 2025b
Phage-CRISPR–Cas9 (CRISPR-Phage)	Phage delivers CRISPR machinery → targeted DNA cleavage of resistance or virulence genes → strain-specific killing	MDR pathogens, microbiome modulation, plasmid curing	Highest specificity; eliminates only targeted strains; reverses antibiotic resistance by cutting resistance genes	Requires engineering; delivery efficiency varies; bacteria may evolve anti-CRISPR systems; regulatory and biosafety concerns	Lam et al., 2021; McNeilly et al., 2021; Balcha and Neja, 2023; Chaudhary et al., 2025
Phage-enzymes (Endolysins, Depolymerases)	Enzymes degrade biofilm matrix and capsules; facilitate phage access to hidden bacterial cells	Gram-positive infections, biofilms, encapsulated bacteria	Immediate killing; works even on dormant cells; effective against thick biofilms	Limited delivery *in vivo*; may require repeated dosing; immune neutralization of enzymes	Husain et al., 2025
Phage-nanocarriers/nanocarrier-guided delivery	Phage provides host specificity; nanocarrier delivers drug/AgNP/antibiotic with controlled release	Targeted drug delivery, deep biofilms, intracellular infections	High specificity; controlled release; multifunctional platform (imaging + therapy)	Complex design; cost; stability issues; large-scale production challenges	Ahmed et al., 2025
Phage cocktails (phage-Phage)	Broader host range; reduced resistance evolution; complementary receptor targeting	Polymicrobial infections, rapidly mutating pathogens	Reduces bacterial escape; broad coverage; high safety	Phage–phage interference; production complexity; regulatory challenges	Yoo et al., 2024

## 14 Phage silver nanoparticle conjugate therapy

Silver nanoparticles (AgNPs) are metallic particles of silver in the nanoscale size, which typically varies from 1 to a few hundred nanometers. In this size, silver can be considered to possess physicochemical properties as it has an enormously high surface to volume ratio. It results in the enhancement of surface reactivity, adjustable surface charge and a controlled release property for silver ions (Yaqoob et al., 2020). Silver nanoparticles can be synthesized by numerous means, including traditional, chemical and physical pathways as well as “green” syntheses. In this pathway, biological extracts are used to reduce silver ions and cap the resultant particles for improved biocompatibility (Vadakkan et al., 2024). The particle size, shape and capping agents jointly determine stability, ion release kinetics and biological interaction. These parameters are crucial when engineering AgNPs for antimicrobial use (Rodrigues et al., 2024). AgNPs function as antibacterial agents through several overlapping modes of action, which make them potentially useful with broad efficacy against bacteria and bacterial communities like biofilms. Upon contacting with the bacterial cell, AgNPs initially interact with a cell envelope where it binds onto and disrupt the membrane surface. This interaction can lead to increased membrane permeability and structural damage that affects the cell integrity. AgNPs release Ag^+^ ions which diffuse into the periplasm and cytoplasm. They attach to protein and enzyme thiol groups, leading to the inactivation of major metabolic pathways and blocking respiration as well as membrane transport (Abdelsattar et al., 2021). In addition, exposure to AgNPs triggers oxidative stress by generating Reactive Oxygen Species (ROS), which cause damage to DNA, proteins, and lipids. Ag^+^ ions can also bind to nucleic acids and ribosomes, effectively blocking replication and translation. Proteomic studies also show the disruption of cellular protein networks (Miethke et al., 2021). Silver nanoparticles initiate a multi-targeted destruction, and any type of mutation that bacteria cannot resist.

Despite these features, many essential constraints and safety concerns for immediate clinical translation. Recent reports detail the emergence of silver tolerance or resistance in clinical isolates, sequestration proteins, genetically encoded efflux pumps, or other detoxification machineries that decrease bacterial sensitivity to silver, particularly in nosocomial pathogens, such as *Acinetobacter baumannii* (McNeilly et al., 2025). Silver nanoparticles engineered with bacteriophages, represent a strong and powerful augmentation of antimicrobial strategies. There are three different ways to combine the bacteriophages with the silver nanoparticles. The co-application, or cocktail approach, is the easiest and quickest to implement, where phages and AgNPs are delivered to the site of infection together. The two agents can act independently. The nanoparticles weaken the membranes and expose the underlying bacteria to the phages. The bacteriophages bind to the specific receptors, inject their genomes, and proceed to replicate, ultimately lysing the host bacterial cell (Guo et al., 2021). The combined pressure of the two agents results in faster and more thorough clearance of bacteria as compared to what either agent can achieve on their own. Biofilms can make bacteria resistant to bacteriophage-based therapy. AgNPs may have an influential effect on phage infection by depolymerizing the biofilm matrix or changing properties of membranes. When phages are engineered to carry the nanoparticles, the nanoparticles can combat biofilm-embedded cells. They can partially degrade the extracellular polymeric substance encasing the biofilm and allow phages to access and diffuse more readily (Zuñiga-Miranda et al., 2024; Wdowiak et al., 2025a).

Another approach involves the physical conjugation of AgNPs to phage particles through chemical linkers or the presentation of nanoparticle-binding peptides on the phage capsid. In this type of engineered phage-AgNP complexes, the phage acts as both a targeting ligand and a delivery vehicle. The phage adsorbs to its host bacterium; the nanoparticles gain access to the cell surface and concentrate silver. The phage provides intracellular silver and membrane-destabilizing elements that can assist to kill bacteria. This effect enhances the potential of silver where it can be lethal. Recent studies have shown that conjugates in this order kill more target bacteria and improve biofilm clearance compared to unconjugated mixtures. Bacteriophages can also be used with AgNPs in a different way, as precision scaffolds to direct nanoparticle-based carriers to specified bacterial strains. In this phage-guided nanocarrier approach, phages are attached to or displayed on the surface of the nanoparticles. The phage provides host specificity and docking ability, while the nanoparticle serves as a reservoir of antimicrobial. They improve the imaging capacity and regulate the release of therapeutic agents. This strategy enables targeted killing of specific bacteria in complex mixtures of microorganisms and has been suggested as a route to the development of antimicrobials (Ahmed et al., 2023; Tanveer et al., 2024). The mixed phage-AgNP system causes two kinds of stresses to bacteria. Silver nanoparticles damage outer membrane of the bacteria and cause metabolic homeostasis whereas phages run an intracellular process of replication leading to lysis. These phages replicate inside the bacteria and able to expose the intracellular machinery of the bacterial cell to silver reactivity and trigger cell death more rapidly. Such mechanisms can eliminate bacteria more effectively than phage monotherapy and create an increased evolutionary barrier to resistance of bacteria. Bacteria must be able to acquire resistance to both particle-delivered chemical stress as well as phage infection simultaneously in order to survive. Figure 3b illustrates phage–silver nanoparticle conjugate therapy, in which phages are chemically or electrostatically bound to AgNPs. The phage component confers targeting specificity and facilitates adherence to the bacterial surface, while the silver nanoparticles provide potent antimicrobial activity through membrane disruption, oxidative stress generation, and interference with metabolic enzymes. The synergistic mechanism results in enhanced killing even of biofilm-associated or resistant bacteria.

To realize the therapeutic potential of phage-AgNP combinations, several practical directions should be prioritized in future research. Nanoparticle formulations must be rationally optimized and particle size, shape, and surface functionalization should be chosen to maximize antimicrobial potency while preserving phage integrity and minimizing host toxicity. Phage-NP compatibility screening is also essential and the applications should be directed where localized high-efficacy treatment is most needed, such as chronic wound biofilms, implant-associated infections and surface decontamination (Zuñiga-Miranda et al., 2024). Compatibility of nanoparticle preparations with phage infectivity must be established for this combination to work effectively. Certain AgNP preparations, based on particle chemistry and surface coatings, will inactivate phages or interfere with receptor recognition, and capping agents and conjugation chemistries. Therefore, these conjugates need to be optimized carefully for preservation of phage viability (De Plano et al., 2025). From a safety perspective, systemic exposure to silver nanoparticles is a matter of concern for cytotoxicity, inflammatory response and environmental accumulation. As a result, clinical applications will eventually move toward localized distribution, such as wound closure or catheter locks (Rodrigues et al., 2024).

## 15 Phage enzyme therapy

Phage enzyme therapy is a therapeutic strategy that employs enzymes from bacteriophages that lyse bacteria and disrupt the protective layers around them (Fujimoto et al., 2024; Islam, 2024). It does not require complete phage particles to infect and replicate. Phage enzymes have the most rapid bactericidal action. They can bypass most of the limitations of phage monotherapy, such as receptor-based resistance (Nakonieczna et al., 2024). Numerous studies show that enzymes encoded by phages can damage bacterial cells and destroy biofilms and can be altered to become more target specific antimicrobials (Yehl et al., 2019; Azeredo et al., 2021). They can kill bacteria in adverse situations where phages cannot diffuse or infect efficiently, such as in wounds, dense biofilms, or complex infections. Their specificity is a major advantage. They lyse only the target pathogens without causing any disruption of beneficial microorganisms and the host cells (Stone et al., 2022). Enzymes from different classes, especially endolysins are the most popular for degrading the peptidoglycan layer of the bacterial cell wall. Phage endolysins can be engineered for greater potency, host specificity and stability (Hassannia et al., 2024). Phage derived enzymes include depolymerases that break down exopolysaccharides, capsules, lipopolysaccharide O-antigens, and components of biofilm matrices. The polysaccharide layers on the surface of the bacteria are attacked and the bacteria are susceptible to immune cells and antibiotic clearance (Topka-Bielecka et al., 2021). The immediate structural destabilization causes the lysis of the bacteria. Endolysins induce the formation of pores in the peptidoglycan layer causing osmotic lysis and death of the bacterial cell (Shahrour et al., 2025). Depolymerases can remove the extracellular matrix and biofilm polymers and reveal the underlying cells and make the susceptible to the immune responses and other antimicrobials. As a result of the biofilm degradation, the phage-derived enzymes allow higher penetration by antibiotics, phages, and immune cells (Chen et al., 2022). Figure 3c shows phage enzyme therapy, involving the use of isolated phage lysins, endolysins, and depolymerases that degrade peptidoglycan or exopolysaccharides of the bacterial cell wall. Because these enzymes are capable of functioning externally, they may rapidly eliminate bacteria without phage replication and easily break down biofilms and capsule barriers, which usually protect clinical isolates. These enzyme-based therapies also have some restrictions. Their stability, bioavailability, and the immunogenic reactions caused by them will need to be addressed to prolong their activity (Danis-Wlodarczyk et al., 2021). Enzyme design can also constrain the range of bacterial species targeted which complicates their quick deployment in a clinical setting. However, with developments in protein research and development, phage enzyme therapy has the potential to be an essential tool in combating antibiotic-resistant bacterial infections.

## 16 CRISPR-Cas9 mediated phage therapy

The Clustered Regularly Interspaced Short Palindromic Repeats (CRISPR) and associated Cas (CRISPR-associated) proteins constitute an adaptive immune system, which is naturally found in many bacteria and archaea. Among these, CRISPR-Cas9, derived from *Streptococcus pyogenes*, is the most well-characterized and widely used tool for genome editing (Lemaire et al., 2022). CRISPR-Cas9 protects bacteria against invading genetic elements such as bacteriophages and plasmids by recognizing and cleaving their foreign DNA sequences (Balcha and Neja, 2023). The system is composed of two main components, a Cas9 endonuclease and a guide RNA (gRNA). The gRNA is a short RNA molecule that directs Cas9 to a specific DNA sequence through base-pairing complementarity (Hu et al., 2023). CRISPR-Cas9 binds to the bacterial genome and induces a double-strand break (DSB) at the target site. It can cause either disruption or modification of the targeted gene (Xue and Greene, 2021). This property is useful for combating antibiotic resistance. Antibiotic resistance has become one of the most significant global health crises. CRISPR-Cas9 can be a target-specific alternative as it can inactivate the resistance genes or essential bacterial genes but does not kill pathogenic or beneficial bacteria (Wan et al., 2021). CRISPR-based antimicrobials can remove specific bacterial populations and restore microbial balance (Palacios Araya et al., 2021). They can also prevent damage to the beneficial microbiota. This strategy also reduces the selective pressure that usually leads to the development of antibiotic-resistant bacteria. CRISPR-mediated therapy has emerged as a possible next-generation antibacterial approach due to its specific targeting (Amen et al., 2025). They can also be engineered to remove antibiotic resistance plasmids and eliminate virulence factors. It can resensitize bacteria to conventional medicines and restore the efficacy of current antibiotics (Islam et al., 2024b; Saffari Natanzi et al., 2025). Bacteriophages are the natural predators of bacteria. They are perfect carriers for delivering CRISPR-Cas9 components to bacterial cells. These phages have extraordinary host selectivity and innate infection machinery, that allows them to efficiently insert genetic material into bacteria (Yeh et al., 2022). If these phages can be rerouted to infect bacteria by genetically modifying them to carry the CRISPR-Cas9 cassette, they can precisely remove virulent or antibiotic-resistant strains. In this combination therapy, phages serve as CRISPR delivery vehicles (Khambhati et al., 2023).

In this therapy, a guide RNA (gRNA) is designed to a specific bacterial gene of interest, such as, a bla or mecA gene, a resistance gene, or other genes that are virulence factors and pathogenic (Li et al., 2022; Khambhati et al., 2023). Once the CRISPR-Cas is constructed, that contains cas9 nuclease and the relevant gRNA, it is integrated into the phage genome. This phage can be made through different molecular methodology, such as homologous recombination, phage rebooting systems and synthetic genome assembly (Pires et al., 2021; Zheng et al., 2023; Ipoutcha et al., 2024). The modified phage now has the ability to express the CRISPR-Cas9 machinery. In this step, the engineered phage infects the bacteria and injects the CRISPR-Cas9 system with its genetic material and the cas9 and gRNA into the bacterial cytoplasm. It targets and cleaves the bacterial chromosome or plasmids at specific regions. The gRNA complex acts as a guide for the cas9 to target the specific DNA for a cut. Cleavage of DNA damages vital genes or resistance-conferring plasmids and causes cell death (Uribe et al., 2021). This effective gene targeting allows selective bacterial killing by removing unwanted cells. It causes induced cell death or the loss of resistance genes. The beneficial microbiota remains unharmed. This specificity helps CRISPR-Cas-mediated phage therapy and make it one of the most powerful therapeutic tools (Wang et al., 2023). Figure 3d depicts CRISPR-Cas9-mediated phage treatment, in which modified phages transmit programmable CRISPR-Cas9 machinery to target antibiotic resistance genes or key chromosomal regions in pathogens. Following delivery, Cas9 causes double-strand DNA breaks, resulting in lethal gene disruption and more targeted elimination of resistant strains, virulence factors, or specific bacterial genomes from heterogeneous microbial communities.

## 17 Limitations and future perspectives

Despite their transformative potential, the development and clinical translation of phage-based therapies face several significant hurdles. A primary constraint is the inherent narrow host range of many wild-type phages, which, while ensuring specificity, limits their applicability against diverse or evolving bacterial populations in polymicrobial infections. Furthermore, biofilms present a formidable physical and physiological barrier, as their dense extracellular matrix can inhibit phage penetration and adsorption, shielding resident bacteria. The pharmacokinetics and stability of phage preparations and their derived enzymes including short half-lives, potential for rapid clearance by the reticuloendothelial system, and susceptibility to inactivation in adverse physiological environments remain poorly characterized and pose a challenge for systemic delivery. From a safety and regulatory standpoint, the immunogenicity of phage particles and bacterial enzymes can trigger neutralizing immune responses, potentially reducing efficacy upon repeated administration and causing inflammatory side effects. The technical complexity of certain approaches also presents limitations; for instance, CRISPR-Cas9 delivery requires sophisticated genetic engineering of phage vectors, and the potential for off-target effects or the transfer of genetic material to non-target bacteria warrants careful investigation. Finally, the scalable manufacturing of consistent, high-purity, and genetically stable phage cocktails or engineered conjugates under Good Manufacturing Practice (GMP) standards is a non-trivial logistical and economic challenge that must be overcome for widespread clinical adoption.

Looking ahead, the future of phage-based therapeutics lies in the strategic integration of synthetic biology, materials science, and clinical medicine to overcome current limitations and unlock new functionalities. Key research directions will include the rational design of synthetic phage platforms. Using advanced genetic tools, phages can be engineered with expanded host ranges, enhanced biofilm-penetrating capabilities, and stealth properties to evade the immune system. The functional repertoire of phages will be further augmented by designing them as modular delivery systems for novel cargo, including next-generation antibiotics, photosensitizers for photodynamic therapy, and tailored gene-editing systems beyond Cas9. A major frontier is the development of intelligent combination therapies. Evolutionary-trained phage cocktails, designed to exploit bacterial resistance trade-offs, can be strategically paired with sub-inhibitory concentrations of antibiotics to resensitize resistant strains. Similarly, the synergy between phage-derived depolymerases and standard-of-care drugs offers a promising path to break down recalcitrant biofilms. The translation to clinical applications will require a concerted effort to establish standardized protocols for phage isolation, characterization, and potency assays. Robust pharmacokinetic and toxicology studies are essential to define safe dosing regimens, while well-controlled clinical trials must be conducted to validate efficacy in target indications such as chronic wound infections, ventilator-associated pneumonia, and implant-associated biofilms. Notably although plant virus nanoparticles (VNPs) and virus-like particles (VLPs) exhibit promising biocompatibility and safety, their clinical translation is still more limited than that of bacteriophage-based systems because of issues with cost, long-term evaluation, and regulations.

Finally, the convergence of phage technology with diagnostics paves the way for personalized and predictive medicine. The ability to rapidly identify a pathogen and deploy a tailored phage cocktail or enzyme against it represents the ultimate expression of precision medicine. By addressing the current challenges through interdisciplinary innovation, phage-based platforms are poised to transition from a promising alternative to a mainstream therapeutic option, ultimately reshaping our approach to combating antibiotic-resistant infections and complex diseases.

## 18 Conclusion

The escalating crisis of antimicrobial resistance and the complexities of treating diseases like cancer demand a paradigm shift toward highly specific, adaptable therapeutic platforms. Bacteriophages, once viewed narrowly as natural bacterial predators, have emerged as uniquely versatile vectors at the forefront of this precision medicine revolution. As this review has elaborated, the potential of phage-based systems extends far beyond traditional lytic therapy into a sophisticated toolkit for bimodal intervention. Through bioengineering, phages can be transformed into targeted nanoscaffolds capable of delivering diverse cargo from conventional cytotoxic drugs and immunomodulators to advanced genetic editors like CRISPR-Cas directly to diseased cells, thereby maximizing efficacy while minimizing off-target effects. The convergence of phage biology with nanotechnology and synthetic biology has given rise to powerful combination strategies. The integration of phages with silver nanoparticles merges the specificity of viral targeting with the broad-spectrum, disruptive power of metallic ions, creating a synergistic force effective against resilient biofilms. Similarly, the deployment of phage-derived enzymes, such as endolysins and depolymerases, offers a rapid, replication-independent mechanism to dismantle bacterial cell walls and biofilm matrices, sensitizing pathogens to conventional treatments. Most profoundly, the engineering of phages to carry CRISPR-Cas systems represents the apex of this precision approach, enabling the programmable elimination of antibiotic resistance genes or the selective destruction of pathogenic strains based on their genetic signature. However, the translational journey of these platforms from laboratory innovation to clinical mainstay is not without hurdles. Key challenges include optimizing the stability and bioavailability of phage-enzyme conjugates, mitigating potential immunogenicity, navigating the complex regulatory pathways for genetically modified biologics, and scaling up manufacturing for widespread clinical application. Future research must focus on refining delivery systems to overcome physiological barriers, conducting robust preclinical and clinical trials to validate safety and efficacy, and developing intelligent, evolution-informed cocktail designs that preempt resistance. The amalgamation of phages' innate bactericidal properties with the principles of bioengineering and nanotechnology positions them as transformative agents in the therapeutic landscape. By harnessing their dual capability as both therapeutics and targeted delivery vehicles, phage-based platforms hold the transformative promise to usher in a new era of personalized, effective, and precise medicine for a broad spectrum of infectious, oncologic, and genetic diseases.
