# Correction: Phage enabled precision drug delivery: dual function platforms for therapeutics and genetic cargo transport

**DOI:** 10.3389/fmicb.2026.2036783

**Published:** 2026-10-29

**Authors:** Md. Sharifull Islam, Jie Fan, Marshia Ahmed Vabna, Nabila Haque, Sawda Binte Monir, Yikui Li, Ishatur Nime, Mrityunjoy Acharjee, Fan Pan

**Affiliations:** 1 Center for Cancer Immunology, Institute of Biomedicine and Biotechnology, Shenzhen Institute of Advanced Technology (SIAT), Chinese Academy of Sciences (CAS), Shenzhen, China; 2 Department of Microbiology, Stamford University Bangladesh, Dhaka, Bangladesh; 3 Department of Pathology, School of Basic Medicine, Henan University of Science and Technology, Luoyang, China; 4 Department of Microbiology, Jahangirnagar University, Savar, Bangladesh; 5 Laboratory of Environment Correlative Dietology, College of Food Science and Technology, Huazhong Agricultural University, Wuhan, Hubei, China; 6 Faculty of Pharmaceutical Sciences, Shenzhen University of Advanced Technology (SUAT), Shenzhen, China

**Keywords:** antimicrobial resistance, combination therapy, nanotherapy, phage delivery, phage engineering, precision medicine

The affiliation “Faculty of Pharmaceutical Sciences, Shenzhen University of Advanced Technology (SUAT), Shenzhen, China” was omitted from the paper.

This affiliation has now been added as the only affiliation for author [Fan Pan].

The original version of this article has been updated.

